# Extraction Equilibrium of Indium(III) from Nitric Acid Solutions by Di(2-ethylhexyl)phosphoric Acid Dissolved in Kerosene

**DOI:** 10.3390/molecules17010408

**Published:** 2012-01-04

**Authors:** Hung-Sheng Tsai, Teh-Hua Tsai

**Affiliations:** Department of Chemical Engineering and Biotechnology, National Taipei University of Technology, Taipei 10608, Taiwan; Email: hungsheng.tsai@gmail.com

**Keywords:** extraction, equilibrium, indium, di(2-ethylhexyl)phosphoric acid, D2EHPA

## Abstract

The extraction equilibrium of indium(III) from a nitric acid solution using di(2-ethylhexyl) phosphoric acid (D2EHPA) as an acidic extractant of organophosphorus compounds dissolved in kerosene was studied. By graphical and numerical analysis, the compositions of indium-D2EHPA complexes in organic phase and stoichiometry of the extraction reaction were examined. Nitric acid solutions with various indium concentrations at 25 °C were used to obtain the equilibrium constant of InR_3_ in the organic phase. The experimental results showed that the extraction distribution ratios of indium(III) between the organic phase and the aqueous solution increased when either the pH value of the aqueous solution and/or the concentration of the organic phase extractant increased. Finally, the recovery efficiency of indium(III) in nitric acid was measured.

## 1. Introduction

Indium is a crucial metal in the electronics industry. For example, indium tin oxide (ITO) is widely used and plays an important role in the LCD and solar energy industries. However, indium is scarce in the Nature, and is estimated to be 0.05 part per million in the continental crust. In 2009, the average annual price of indium was 500 USD/kg. Currently, the price is around the 565 USD/kg [1]. Thus, the development of technology for recovery of indium from waste material will be critical needed to meet the demand from the industry. In developing metal recovery technology, solvent extraction will be the essential technique and a key step in hydrometallurgy [2,3].

Acidic organophosphorus compounds are very important extractants in the solvent extraction of indium [4,5,6,7]. The indium can be extracted from nitric acid using di(2-ethylhexyl)phosphoric acid (abbreviated as D2EHPA or simply HR), 2-ethylhexylphosphonic acid mono-2-ethylhexyl ester (PC-88A), and di(2,4,4-trimethylpentyl)phosphinic acid (Cyanex 272) dissolved in toluene [8]. These studies indicated at low loading ratios that the complexes of all extractants were InR_3_(HR)_3_. The extraction of indium from a nitrate solution could be done by using di(2-ethylhexyl)phosphinic acid (PIA-8) dissolved in toluene [9]; and indium can be extracted using D2EHPA dissolved in octanoic acid from a sulfate solution [10]. Both studies obtained complex compositions similar to that of Inoue’s study [8]. The complex composition of indium, InR_3_(HR), was also observed by using D2EHPA dissolved in methyl isobutyl ketone (MIBK), a polar solvent, to extract indium from a nitrate solution [11]. Using kerosene as a diluent to extract indium from a chloride solution demonstrated that when the organic phase was at low loading ratio, the complexes of D2EHPA and PC-88A were both InR_3_(HR), whereas the complex of PIA-8 was InR_3_Cl(HR)_2_ [12]. In the case of extraction of indium from a sulphuric acid solution using D2EHPA/kerosene solution [13], it was found when the concentration of H_2_SO_4_ was between 0.5 to 4.0 M, the complex was InR_3_(HR)_3_, and when concentration dropped below 0.5 M, the complex became In_2_R_10_H_4_. It was also found at high indium concentrations, complexes with 1:3 metal:reagent would be formed. When using kerosene as a diluent, D2EHPA possessed a better indium extraction efficiency than PC-88A did [14].

For the separation of metals, D2EHPA is an effective extractant in the hydrometallurgy process because of its high selectivity for many systems, and its chemical stability, and extremely low solubility in acidic aqueous solutions [15,16]. The results of the extraction of indium [5], indicated that kerosene has strong effects as a dilute to the extraction of indium. Therefore, D2EHPA as the extractant and kerosene as the diluent were selected in this study.

The purpose of this study is to determine indium extraction equilibrium, using D2EHPA as an extractant and kerosene as a diluent to extract indium(III) from a nitric acid solution at 25.0 ± 0.2 °C. In order to obtain a complete understanding of this system, the extraction of indium(III) is studied by graphical and then verified by the numerical analysis. The recovery efficiency at a fixed pH of aqueous phase will be determined by varying the concentrations of D2EHPA.

## 2. Results and Discussion

### 2.1. Extraction Equilibrium of Indium

In this study, indium(III) was extracted using D2EHPA. According to the potential-pH equilibrium diagram [18], when the pH value was below 2.5, the indium ions in an aqueous solution were primarily in the form of In^3+^. However, when the pH value was between 1 and 3, indium ions in the aqueous phase were also present in the form of In(OH)^2+^ [5]. Therefore, this study maintained a pH value less than 1 during the aqueous phase to maintain the stability of the system.

When the D2EHPA loaded in the organic phase was near to saturation, the metal-D2EHPA complexes continued to react and formed polymeric species. In the presence of free D2EHPA molecules and ethylene glycol, these polymers depolymerized [19]. Therefore, under assumption that the extraction of indium(III) with D2EHPA causes the formation of an *m*-merized complex, the extraction reaction could be expressed as:

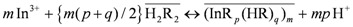
(1)

The stoichiometric extraction equilibrium constant could be expressed as:

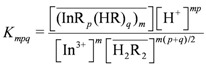
(2)
where the concentration with bar signifies that the species are in the organic phase.

The distribution ratio of indium was defined as:

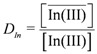
(3)


**Figure 1:**
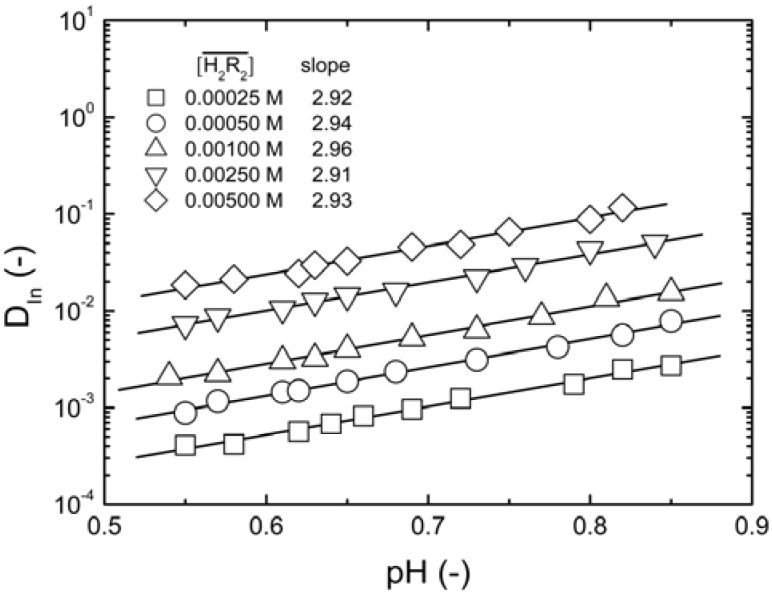
Relationships of log *D_In_ vs.* pH at equilibrium with various D2EHPA concentrations in kerosene at 25 °C. [In^3+^]_t_ = 0.020 to 0.15 kmol/m^3^.

Substituting Equation (2) into (3) we obtain (4):

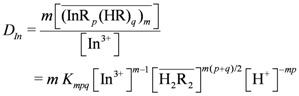
(4)



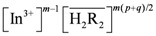
 remained almost unchanged when D2EHPA was present at a low distribution ratio and its concentration in the organic phase varied insignificantly. Therefore, mp can be determined from the slope by plotting log *D_In_ vs.* pH. Figure 1 showed the impact of the pH on the distribution ratio when extracting indium(III) with various concentrations of D2EHPA from the nitric acid aqueous solutions. 

The slope of the straight lines was 3, that is *mp =* 3. Therefore, Equation (4) can be simplified to:

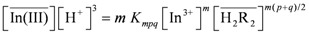
(5)


**Figure 2:**
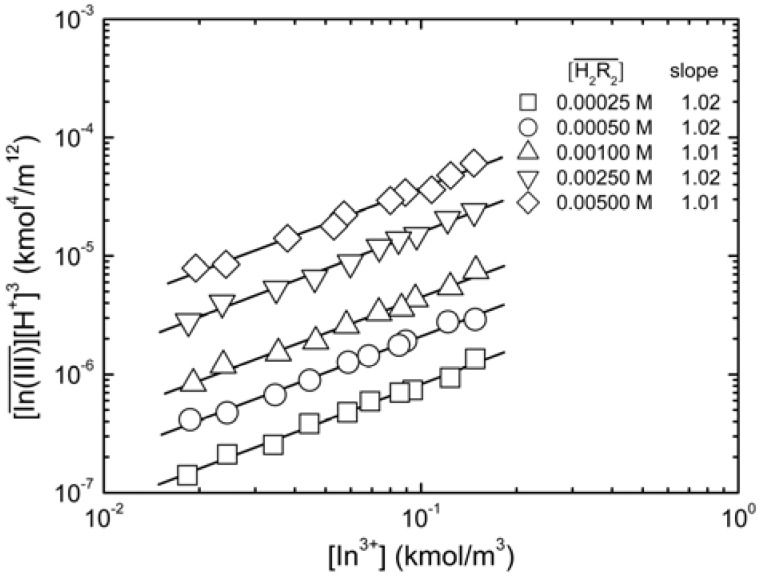
Log-plots of 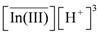 *vs.* 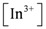 at equilibrium with various D2EHPA concentrations in kerosene at 25 °C. [In^3+^]_t_ 0.020 to 0.15 kmol/m^3^.

When the D2EHPA concentration in the organic phase remained unchanged, the aggregation degree of complexes of indium(III) and D2EHPA in the organic phase, *m*, can be identified by plotting 
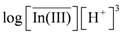
 *vs.* 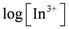. As shown in Figure 2, the slope of the straight lines was 1, that was *m =* 1. Therefore, *p* = 3, and Equations (2) and (4) become:

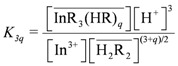
(6)
and:

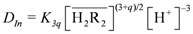
(7)


Reorganizing Equation (7) we obtain:

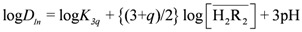
(8)


Next, we assume that only species of the type InR_3_(HR)*_q_* are formed during the organic phase. Thus, plotting log(*D_In_*[H^+^]^3^) *vs.* log[H_2_R_2_] gives (3 + *q*)/2 and logK*_3q_* from the slope and the intercept of the straight respectively.

**Figure 3:**
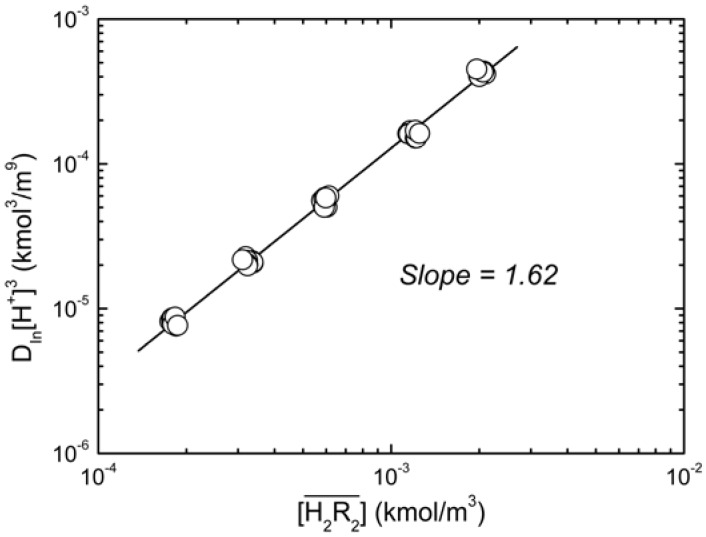
Log-plots of *D_In _*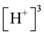 *vs.* 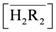 at equilibrium with various D2EHPA concentrations in kerosene at 25 °C. 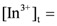 0.020 to 0.15 kmol/m^3^, slope = 1.62.

As shown in Figure 3, the slope of the straight line was 1.62, and *q* = 0.24. Because *q* had a considerably small value, it can be assumed that *q* = 0. The type of species in the organic phase was 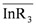; therefore, Equation (3) can be expressed as:

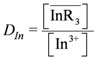
(9)


By using Equations (6) and (9), the equation can be expressed as:

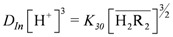
(10)
or:

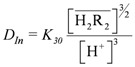
(11)

**Figure 4:**
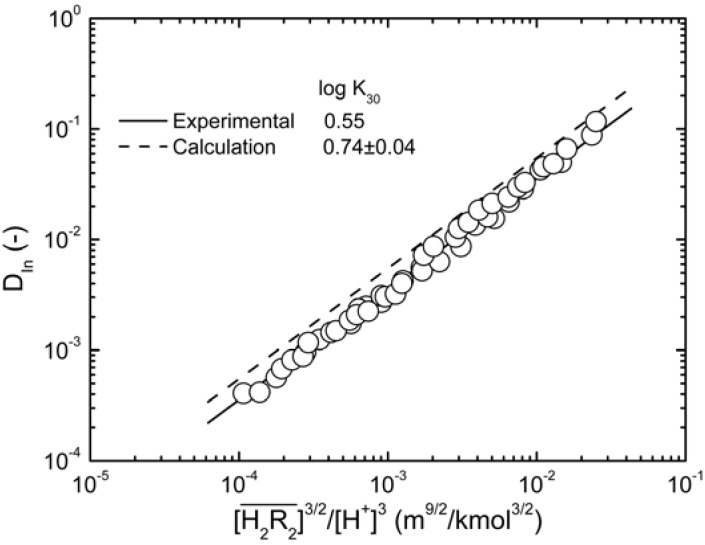
Log-plots of *D_In_ vs.* 
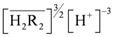
 at equilibrium with various D2EHPA concentrations in kerosene at 25 °C. 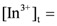 0.020 to 0.15 kmol/m^3^.

Plot log *D_In_ vs.* 
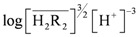
. When the slope is 1, the intercept was log *K_30_*. As shown in Figure 4, the intercept for the straight line is 0.55, that was *K_30_* = 3.55 (kmol/m^3^)^3/2^.

### 2.2. Reconfirmation of Extraction Equilibrium Formation by Computer Analysis

To verify the complex composition and equilibrium constants of the extraction equilibrium, the LETAGROP-DISTR program was used in this study to perform a numerical treatment on data of the four-component system [2,3,20]. The numerical analysis was performed to verify the results of graphical analysis. For the calculation process, to enable the computer to search for the optimal equilibrium constants, the minimized sum of the squared errors was defined as:

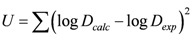
(12)
where *D_exp_* and *D_calc_* were the measured and program-calculated distribution ratios, respectively. The solute concentrations inputted into the program included pH, In^3+^, NO_3_^−^, and H_2_R_2_, to solve the mass balance equations. The acquired best model was the result with minimization of the function *U*. The lowest mean standard deviation σ(log *D*) was then defined as:

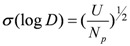
(13)
where *N_p_* represents the degree of freedom, *i.e.*, the number data points.

The equilibrium constants of D2EHPA between the aqueous phase and organic phase were required for the program, as shown in Table 1. The program-calculated *U*_min_ shown that the optimal value for *q* was 0. Therefore, the optimal (*p*,*q*) was (3,0), that was, the complex composition is 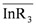. This result was consistent with that obtained from graphical treatment, where the calculated equilibrium constant was log *K_30_* = 0.74 ± 0.04 (the error provided corresponds to 3s(log *K*)). Subsequently, the distribution ratio was calculated as shown in Figure 4, and compared with the experimental value. The results demonstrate that the calculated and the experimental values were very close.

The mass balance equation of 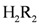 is:

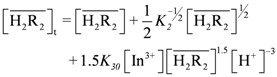
(14)


**Table 1:** The equilibrium constants of the different D2EHPA species [21].

Reaction	Constant
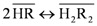	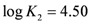
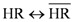	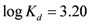
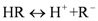	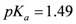

### 2.3. Complexation of Indium in Nitrate Solutions

**Figure 5:**
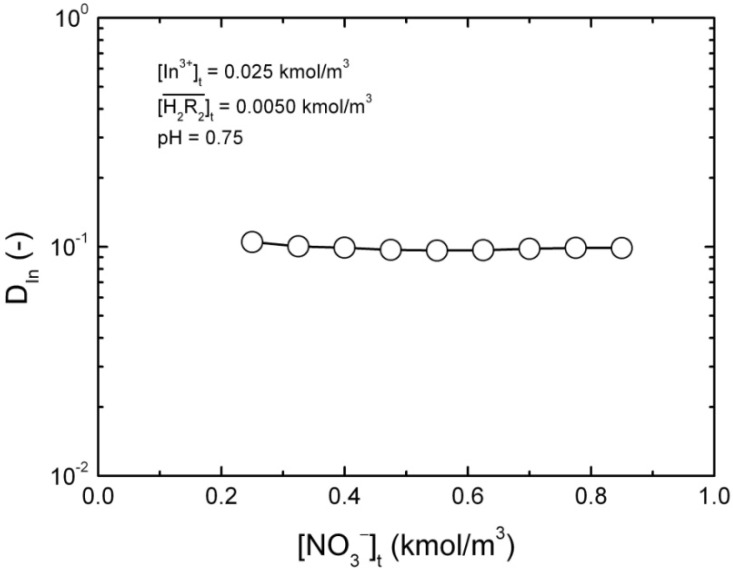
Effect of concentration of nitrate on the extraction of indium with D2EHPA in kerosene at 25 °C.

Experiments were conducted to study the effect of nitrate ions concentration on the extraction distribution ratio of indium. Figure 5 shows that there was no significant effects to the distribution ratio of indium when the concentration of nitrate increased.

From the results of extraction equilibrium experiments, it was found that three protons would be released to aqueous phase during the D2EHPA extraction of indium, *i.e.*, indium would react with D2EHPA in the form of In^3+^. However, indium and nitrate ions in aqueous phase still will form complex reactions when in contact with each other; therefore, the stability constants of In(NO_3_)^2+^ and In(NO_3_)_2_^+^ were defined as:

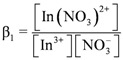
(15)
and:

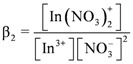
(16)
Log *β_1_* and log *β_2_* are 0.18 and -0.31, respectively [22]. [NO_3_^−^] can be calculated using the mass balance equation of the nitrate concentration, as follows

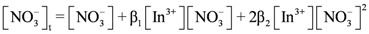
(17)


**Figure 6:**
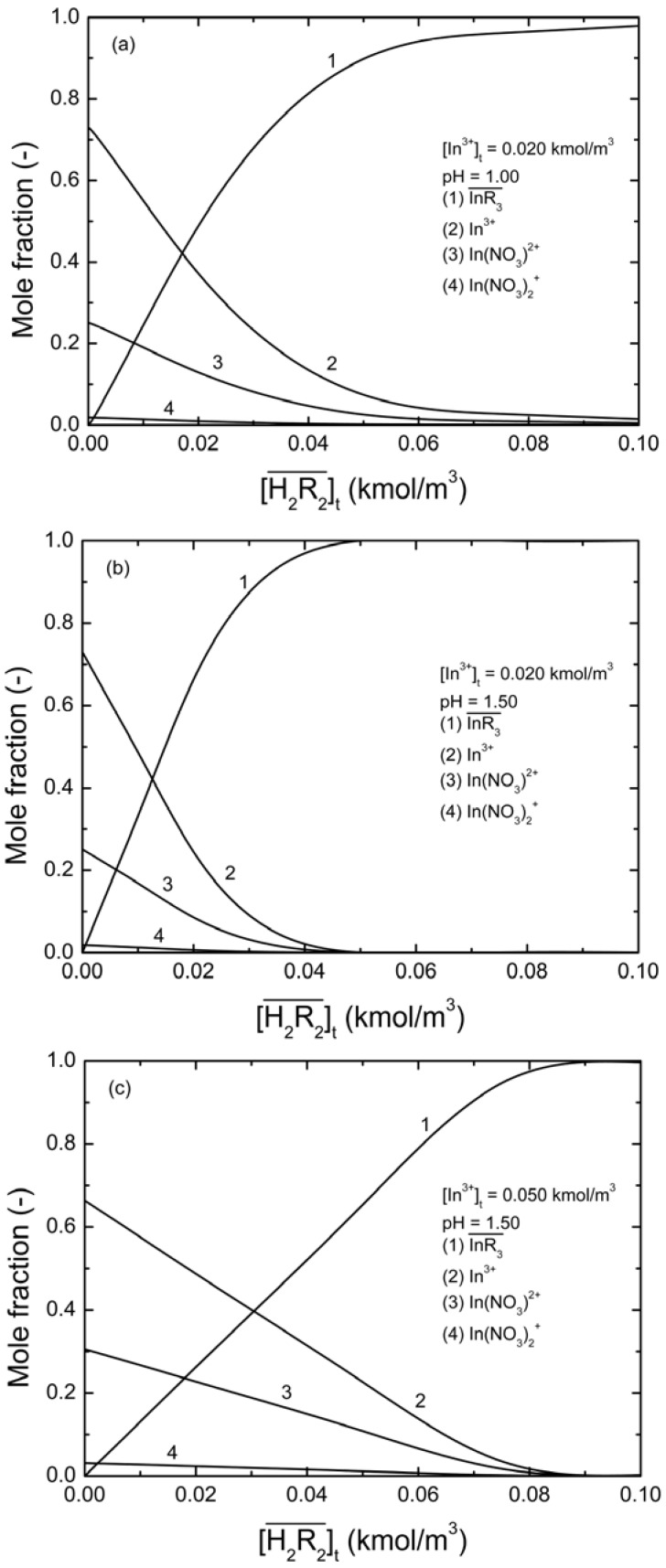
The mole fractions of indium ions and their complexes as a function of the total D2EHPA concentration at two different total concentrations of indium and two pH values.

Figure 6 shows the distribution of the molar fraction of indium ion at different D2EHPA concentrations under the effects of the complex reactions of indium and nitrate ions.

As indicated in the figure, the 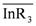 concentration increased as the total concentration of D2EHPA increased; while the concentrations of In^3+^, In(NO_3_)^2+^, In(NO_3_)_2_^+^ decreased. Figure 6a,b show that, under the same indium ions concentrations, the higher pH of aqueous phase, the better extraction efficiency of D2EHPA for indium(III). In other words, it is easier to form 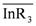 in the organic phase. Figure 6b,c indicated the extraction amount of indium by D2EHPA can be increased by raising the indium ions concentration in the aqueous phase at the same pH. However, only at higher D2EHPA concentration, indium(III) can be extracted totally from the aqueous phase. Table 2 displays the different equilibrium constants used in this study and discussed in literatures.

**Table 2:** The literature equilibrium constants of 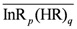 extracted by D2EHPA.

Aqueous phase	Temp.(°C)	Diluent	log *K_pq_*			Reference
			(3,0)	(3,1)	(3,3)	
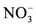	25 ± 0.2	Kerosene	0.55			This work
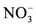	30	Toluene			3.20	[8]
*I* = 6.0 M 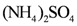	25 ± 0.1	Octanoic acid			2.3 ± 0.2	[10]
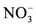	25 ± 0.2	MIBK		4.21		[11]
*I* = 1 M 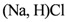	25	Kerosene		3.98		[12]

### 2.4. Recovery Efficiency of Indium

**Figure 7:**
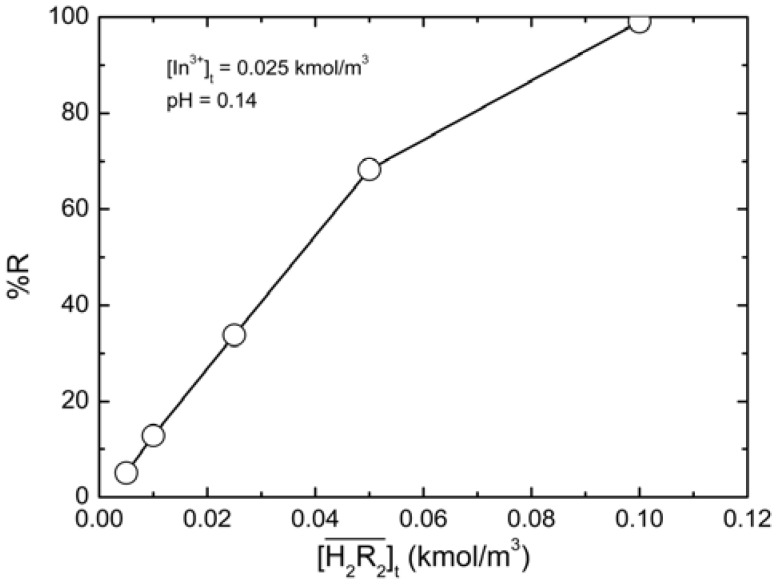
Relationship between the recovery efficiency of indium(III) (%R) and 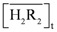 at equilibrium with various D2EHPA concentrations in kerosene at 25 °C. 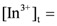 0.025 kmol/m^3^, pH = 0.14.

As a common industrial practice, indium-containing solid waste was treated with acid to dissolve indium prior to extraction and separation. The result was an indium-containing strong acid solution. From Equation (1), the stronger the acidified solution, the poorer the extraction of indium. However, as shown in Figure 7, the experimental results indicated that D2EHPA still had significant extraction effects even in the acid aqueous solution. The effect of extractant concentration on the recovery efficiency was studied. The recovery efficiency of indium(III) (%R) *vs.* 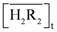 was plotted in Figure 7. As the D2EHPA concentration increased, the recovery efficiency of indium(III) increased accordingly; when 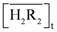 = 0.10 kmol/m^3^, the recovery efficiency could be as high as 99.09%.

## 3. Experimental

### 3.1. Reagents and Solutions

Stock indium solution of 0.5 kmol/m^3^ was prepared by dissolving metal (99.99% purity) in nitric acid, and diluted with distilled water. Prior to conducting the extraction experiments, an appropriate amount of stock solution was mixed with distilled water and 0.5 kmol/m^3^ nitric acid solution to prepare solutions with various pH and metal concentrations. The di(2-ethylhexyl)-phosphoric acid was obtained from Daihachi Chemical Industry Co., Ltd., Osaka, Japan. Potentiometric titration was performed using NaOH in an ethanolic medium to verify its purity as 95%. The precipitates from a copper-D2EHPA complex were separated from toluene and acetone solutions and then dissolved in a toluene and 4 kmol/m^3^ sulfuric acid solution according to the D2EHPA purification procedure [23]. The purity of D2EHPA could be as high as 99.5% after the purification. Kerosene (provided by Chinese Petroleum Co., Taiwan) was used as a diluent. The kerosene was washed three times using 98% sulfuric acid at 1/5 volume of that kerosene, and then washed with distilled water until the solution became neutral [17]. All the other inorganic chemicals used in this study were analytical reagent grade and provided by Shimakyu’s Pure Chemical Industry, Ltd., Osaka, Japan.

### 3.2. Procedure

Equal volumes of organic and aqueous solutions (20 mL), were mixed in a glass flask equipped with ground glass stoppers. Then, the mixture was shaken vigorously by a mechanical shaker at 25.0 ± 0.2 °C for 30 min. The preliminary experiments indicated that the extraction process reached equilibrium within 15 min. The concentration of indium ion in the initial aqueous solutions ranged from 0.020 to 0.15 kmol/m^3^. The organic solutions contained 2.50 × 10^-^^4^ to 0.20 kmol/m^3^ of monomeric D2EHPA dissolved in kerosene. The mixture was placed in the thermostat at 25.0 ± 0.2 °C for more than 12 h before performing a two-phase separation.

Following the phase separation, the equilibrium hydrogen ion concentration was measured using a pH meter, and the indium concentration was measured using a GBC SenseAA atomic absorption spectrophotometer (AAS) at a wavelength of 303.9 nm. The indium in the organic phase was stripped with 4 kmol/m^3^ of hydrochloric acid, and the concentration of indium was measured in an acidic solution using AAS. The metal mass balance during the extraction and stripping procedure must be maintained at ±2%. The concentration of free D2EHPA in the organic phase at equilibrium was determined by a mass balance [2,3].

## 4. Conclusions

From the experiments of indium(III) extraction using D2EHPA dissolved in kerosene from a nitric acid solution at 25 °C, it was found that the distribution ratio of indium(III) in the organic phase and the aqueous phase increased when the D2EHPA concentration or the pH value increased. By graphical and numerical analysis, the composition of the complex was determined to be 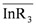, in which the equilibrium constants of experimental and calculated values were log *K_30_* = 0.55 and 0.74 ± 0.04, respectively. No significant effects on the extraction distribution ratio of indium with increased nitrate ions concentration were found. The 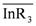 concentration in the organic phase increased as the total concentration of D2EHPA increased, while the concentrations of In^3+^, In(NO_3_)^2+^, In(NO_3_)_2_^+^ in the aqueous phase decreased. As for the metal recovery, when 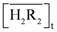 = 0.10 kmol/m^3^, the recovery efficiency of indium(III) could be as high as 99.09%, even when the pH of the solution was 0.14. D2EHPA had significant extraction efficiency for indium in the strong acidic solution.
